# Identifying Plectin Isoform Functions through Animal Models

**DOI:** 10.3390/cells10092453

**Published:** 2021-09-17

**Authors:** Maria J. Castañón, Gerhard Wiche

**Affiliations:** Max Perutz Laboratories, Department of Biochemistry and Cell Biology, University of Vienna, 1030 Vienna, Austria; gerhard.wiche@univie.ac.at

**Keywords:** plectin, mouse models, conditional gene targeting, epidermolysis bullosa, myofibrillar myopathies, vascular permeability, simple epithelia, hemidesmosomes, sarcolemma, neuromuscular synapse

## Abstract

Plectin, a high-molecular-weight cytoskeletal linker protein, binds with high affinity to intermediate filaments of all types and connects them to junctional complexes, organelles, and inner membrane systems. In addition, it interacts with actomyosin structures and microtubules. As a multifunctional protein, plectin has been implicated in several multisystemic diseases, the most common of which is epidermolysis bullosa simplex with muscular dystrophy (EBS-MD). A great part of our knowledge about plectin’s functional diversity has been gained through the analysis of a unique collection of transgenic mice that includes a full (null) knockout (KO), several tissue-restricted and isoform-specific KOs, three double KOs, and two knock-in lines. The key molecular features and pathological phenotypes of these mice will be discussed in this review. In summary, the analysis of the different genetic models indicated that a functional plectin is required for the proper function of striated and simple epithelia, cardiac and skeletal muscle, the neuromuscular junction, and the vascular endothelium, recapitulating the symptoms of humans carrying plectin mutations. The plectin-null line showed severe skin and muscle phenotypes reflecting the importance of plectin for hemidesmosome and sarcomere integrity; whereas the ablation of individual isoforms caused a specific phenotype in myofibers, basal keratinocytes, or neurons. Tissue-restricted ablation of plectin rendered the targeted cells less resilient to mechanical stress. Studies based on animal models other than the mouse, such as zebrafish and *C. elegans*, will be discussed as well.

## 1. Introduction

**Figure 1:**
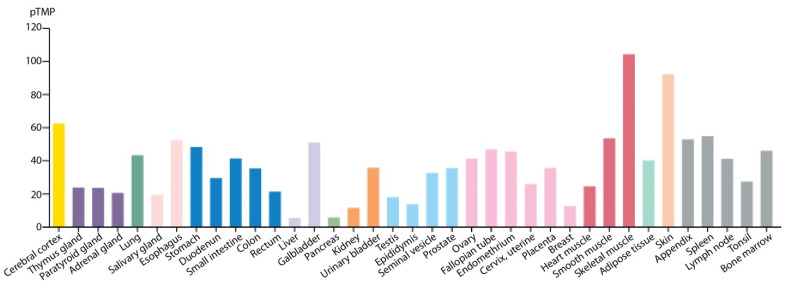
Plectin expression in different tissues. The expression data have been downloaded from the tissue atlas of the Human Protein Atlas (https://www.proteinatlas.org/ENSG00000178209-PLEC/tissue, accessed on 1 June 2021). The data are based on deep sequencing of RNA from 37 major different normal tissues types [9]. TPM, transcripts per million, used as normalization value.

Plectin, a 500 kDa cytoskeletal protein, binds to and interconnects intermediate filaments (IFs) and attaches them to junctional complexes at the cell membrane, different organelles, and inner membrane systems. Plectin also interacts with actomyosin structures and microtubules [1,2]. Additionally, plectin is an integral part of hemidesmosomes. The analysis of the plectin gene revealed an unusual 5′ diversity of differentially spliced plectin transcripts where a dozen alternative first exons encode isoforms with differing short N-terminal protein domains that dictate their expression profile. One variant skipping exon 31 that encodes the entire central rod domain and several interspersed small exons were identified [3,4]. The plectin gene is located on chromosome 8 in humans and chromosome 15 in mice [4,5]. Belonging to the plakin family of proteins, plectin has a multimodular structure with up to seven functional domains. Its variable *N*-termini, defined by short isoform-specific peptides, are followed by an actin binding domain (ABD) and a plakin domain. These isoform-specific *N*-termini are responsible for the differential targeting of plectin to different strategic cellular locations such as the nucleus, mitochondria, the contractile apparatus of muscle cells, hemidesmosomes, focal adhesions, the neuromuscular synapse, and others [6]. The plakin domain is formed by nine spectrin repeats (SRs) and has an src-homology 3 (SH3) domain embedded within the fifth spectrin repeat. The central α-helical domain forms a roughly 200 nm long coiled-coil rod upon dimerization. The *C*-terminal domain consists of six tandemly arranged plectin (or plakin) repeat domains (PRDs) separated by linker regions where the core of the IF-binding domain (IFBD) resides. The tail domain is rich in Gly-Ser-Arg (GSR) repeats [1,2]. Plectin is expressed in a wide variety of cell types and tissues [7,8]. A search for plectin expression in several public databases delivered a quantitative overview, which is summarized in Figure 1. It confirms previous observations indicating that plectin is highly expressed in tissues subjected to great mechanical stress, such as stratified and simple epithelium, skeletal and heart muscle, and blood vessels.

A battery of ultrastructural, biochemical, immunological, and tissue culture assays have provided useful information about the properties and potential functions of plectin, but as the complex interactions among different cell types of tissues and organs cannot be fully reproduced in in vitro systems, plectin functions were studied at the whole-organism level. First insights into the function of plectin in vivo came from patients with mutations in the plectin gene [10]; however, the great part of our knowledge about plectin’s functional diversity has been gained through the analysis of transgenic mice. The mouse was chosen for these studies because mouse and human genomes share about 85% of their genes. Thus, the mouse is the most common animal model to study biological functions and human disease mechanisms. Furthermore, for a long time the mouse was the only species in which it was possible to inactivate individual genes [11] to better understand their functions, and the laboratory mouse has been bred to have a highly homogeneous genetic composition to increase the reproducibility of results. Basically, two strategies have been followed to generate mice depleted of plectin: gene targeting by homologous recombination and conditional gene targeting. Gene targeting by homologous recombination generated transgenic animals where either all plectin isoforms or single isoforms were inactivated in all cell types and tissues (plectin-null and isoform-specific KO mice, respectively). Conversely, conditional targeting, using the Cre/loxP-based gene targeting approach [12], allowed the inactivation of all isoforms expressed in a particular cell type or tissue (e.g., keratinocytes or striated muscle), without interfering with isoform expression in other tissues (conditional KO mice). Other animal models used to study plectin biology are the fish *Danio rerio*, commonly known as zebrafish and the worm *Caenorhabditis* (*C.*) *elegans*. The main advantage of zebrafish as a model organism is that the embryo is transparent and develops outside of the mother in about 5 days, and that its genes can be easily turned on or off. *C. elegans* is a relatively simple organism, has a short life cycle of only two weeks, is transparent throughout its life, and many of its genes have functional counterparts in humans. In contrast, no plectin homolog has been found in *Drosophila.*

## 2. Plectin-Null Mice

The first transgenic animal model generated to establish plectin function in vivo was a KO-mouse [13] created by homologous recombination between the endogenous chromosomal locus and exogenous DNA embedded in the targeting vector [11,14]. By constructing two different targeting vectors, based on the removal of either a large part of the plectin’s central rod domain or exons 2–4, two transgenic lines (plectin-null) were generated. In both cases, homozygous mutant mice die within 2–3 days of birth showing severe skin blistering and structural anomalies in the heart and skeletal muscle. In skin, the blisters are located between the dermis and the upper epidermal layers, arising from the rupture of keratinocytes in the basal cell layer of the epidermis. Structurally, hemidesmosomes (HDs) appeared unaffected, although reduced in numbers; also, their mechanical stability was reduced, as they were missing from areas undergoing blistering and histolysis. In skeletal muscle, there was a visible increase in necrotic muscle fibers; Z-disks and adjacent myofibrils were disorganized, while myocardium was marked by the partial disintegration of intercalated disks [13]. These phenotypes were similar to those reported for the first cases of patients lacking a functional plectin gene [15,16] who suffered from epidermolysis bullosa simplex and muscular dystrophy (EBS-MD). These findings reflected the importance of plectin for the preservation of skin and muscle integrity.

Due to their early death, plectin-null mice were of limited use as a model for EBS-MD. Nevertheless, plectin-null mouse-derived cell cultures of different types became a valuable tool for studying plectin’s functions and mechanism of disease. One example is the rescue of the hemidesmosomal phenotype by forced expression of plectin isoform P1a, but not of P1c, in plectin^−/−^ keratinocytes, which suggested that, specifically, isoform P1a is responsible for the skin phenotype of patients and mice and, thus, has a unique function [17]. The specific association of P1c with microtubules (MTs) was observed for the first time in these cells as well [17]. The phenotype of plectin-deficient fibroblast and astroglial cells showed that the depletion of plectin results in an upregulation of actin stress fibers and focal adhesion contacts pointing towards a role of plectin in counterbalancing actomyosin forces [18].

## 3. Conditional, Tissue-Restricted Plectin KOs

The most common technology to achieve the inactivation of a gene in a timely or tissue-specific manner is the Cre/loxP system. For the system to work, two mouse lines are needed: one carrying the gene to be deleted flanked by loxP sites; the other carrying the Cre recombinase under the control of a promoter active in a specific cell type or tissue, or at a particular developmental stage. After crossing the floxed mouse with a Cre transgenic mouse, the floxed gene is deleted in the cells where Cre is expressed. In the case of plectin, the Cre/loxP system was instrumental in circumventing the early mortality of plectin-null mice and enabled the analysis of plectin functions in adult mice. A plectin floxed transgenic mouse was created to generate a plectin KO in stratified epithelia [19] and has subsequently been used to produce mice with a conditional deletion of plectin in skeletal muscle, Schwann cells, the vascular endothelium, and simple epithelia.

### 3.1. Plectin Ablation in Stratified Epithelia

As plectin is expressed in basal keratinocytes [8], mice carrying the Cre recombinase under the control of the keratin (K) 5 promoter [20] were crossed to plectin floxed mice. The resulting **K5-Cre/cKO** (^plecloxP/loxP^:Krt5-Cre) mice, where the deletion of plectin is restricted to K5-expressing epithelia, died early after birth exhibiting extreme skin fragility manifesting as skin detachment on the fore and hind limbs, large blisters in the upper extremities, arm pits and oral epithelium, focal skin barrier defects, and growth retardation. Apart from the formations of blisters and lesions due to epidermal detachment and their ongoing re-epithelialization, the epidermis of K5-Cre/cKO mice displayed a normal stratified organization and the correct expression of differentiation markers. Proliferation and survival of keratinocytes were unaffected. In the absence of plectin, expression of the hemidesmosomal proteins integrin β4 and BPAG1e, which bind directly to plectin, was slightly reduced, and in the blister areas both proteins were located at the blister floor, indicating disruption of basal keratinocytes at the level of the inner hemidesmosomal plaque. Blisters in the oral cavity, particularly in their palates, most probably impaired food intake, causing starvation and death [19]. To extend the study on the function of plectin in stratified epithelia to adult mice, an inducible KO mouse line was generated by crossing plectin floxed mice with transgenic mice expressing an inducible chimeric Cre-ERT recombinase that can be activated by tamoxifen [21,22]. Although plectin deletion was incomplete, resulting in a mosaic pattern where large patches of epidermis expressing plectin were interspersed with small patches lacking plectin expression, the analysis of the mice demonstrated that the patchwise deletion of plectin rendered the epidermis more prone to trauma [19].

### 3.2. Plectin Ablation in Single Epithelia

Two mouse models have been generated to study plectin in single epithelia. In one, plectin was deleted in the liver [23], in the other, in the intestinal epithelium [24]. The deletion in the liver was achieved by crossing plectin floxed mice with Alb-Cre transgenic mice in which the expression of the Cre recombinase is under the control of the albumin promoter [25]. The resulting **Alb-Cre/cKO** (plec^Δalb^) mice [23] showed efficient excision of the plectin gene in hepatocytes as well as in cholangiocytes, the biliary epithelial cells that line the bile ducts and canaliculi, where plectin is prominently associated with tight and adherens junctions [7]. In both types of cells, the ablation of plectin resulted in aberrant keratin network organization. In hepatocytes, K8/K18 filaments were found scattered throughout the cytoplasm, while biliary epithelial cells had partially lost their apicobasal polarity due to keratin network redistribution. Furthermore, bile ducts and ductules showed an irregular morphology, characterized by elongated ductular lumens that eventually collapse prompting a mild ductular reaction. Misshaped and dilated bile canaliculi with frequent blind end loops and numerous branching points were also observed. Drug-induced liver injury, triggered by feeding the mice 3,5-diethoxycarbonyl-1,4-dihydrocollidine (DDC), revealed that the absence of plectin increases the susceptibility of biliary epithelial cells to DDC intoxication and reduces the capacity of the liver to recover from the injury. Biliary damage upon cholestatic challenge, such as bile duct ligation, resulted in the destabilization of bile ducts due to increasing bile pressure and their subsequent collapse; under similar conditions, K8 formed aggregates in hepatocytes. In response to stress inflicted by exposure to okadaic acid, keratin filaments formed thick filaments bundles that eventually collapsed [23]. Combined, the strong upregulation of plectin and other plakins in models of cholestatic challenge [23,26], the mechanical weakening of the biliary epithelium in the absence of plectin, and the recently reported case of familial intrahepatic cholestasis caused by compound heterozygous mutations in the plectin gene [27], suggest that plectin stabilizes the biliary epithelium, maintains keratin network architecture, and protects the tissue against mechanical stress-induced damage.

Plectin is expressed at high levels in the intestinal epithelium where it forms, together with integrin α6β4, type II HDs. This type of junction attaches the single layer of intestinal epithelial cells (IECs) to the basement membrane [28]. To generate an intestinal epithelium conditional KO mouse, plectin floxed mice were crossed with villin-Cre transgenic mice [29] in which the expression of the Cre recombinase is driven by the promoter of villin, a protein which is highly expressed in the adult intestine. The most significant phenotype of the resulting **Villin-Cre/cKO** (plec^ΔIEC^) mice was the weakening of the intestinal barrier with concomitant loss of its selective permeability, leading to chronic inflammation and colitis [24]. Mechanistically, this is due to the downregulation of the hemidesmosomal and junctional components integrin α6β4, ZO-1, E-cadherin, desmoglein-2 and desmoplakin, resulting in abnormal HD formation, leaky intercellular junctions, and unanchored keratin filaments. As these structures are no longer able to hold the IECs attached to the basement membrane and subjacent lamina propia, or hold the IECs together, an extensive detachment of the IECs ensues, weakening the intestinal barrier [24]. The depletion of plectin in the intestinal epithelium results in a phenotype that is more severe compared to plectin deficiency in the liver [23] but less severe compared to integrin α6β4 deficiency in the intestinal epithelium [30]. This probably reflects that IECs are subjected to greater mechanical stress in the intestine (peristaltic contractility, villous motility) than in the bile duct, and that rudimentary HDs are still formed, whereas the complete absence of HDs is the hallmark of α6-depleted IECs, which no longer express integrin β4 and hardly any plectin in their basal area [30]. Thus, in developing a dysfunctional intestinal barrier, chronic inflammation, and persistent colitis, plectin-cKO mice phenotypically resemble α6-cKO mice. However, in contrast to plectin-cKO mice, the lesions in α6-cKO mice spontaneously degenerate into adenocarcinoma. In humans, plectin expression is significantly reduced in colon biopsies of patients with ulcerative colitis [24].

### 3.3. Plectin Ablation in Endothelial Cells

Endothelial cells lining the vascular vessels are permanently subjected to intense shear stress. As plectin is abundantly expressed in these cells [7] and blood filled blisters have been observed in plectin-null mice [13] as well as in EBS-MD patients, an endothelium-restricted KO mouse, **VE-Cre/cKO** (plec^loxP/loxP^:Cdh5-Cre), was generated to examine the function of plectin in this stress-prone system [31]. To this end, plectin floxed mice were crossed with mice expressing the Cre recombinase under the control of the endothelial-specific VE-cadherin promoter [32]. As quantified in lung endothelial cells, Cre-mediated excision reduced the amount of plectin to ~15% of its normal levels. The main defects observed in these mice were the distorted, discontinuous, and irregularly spaced adherens junctions, particularly at the anterior and posterior cell edges in aortic endothelial cell sheets. Furthermore, an increase in vascular permeability manifesting as increased Evans Blue extravasation was observed in the lung endothelium [31]. To address these phenomenological observations at the cellular level, primary and immortalized cultures from endothelial cells derived from plectin-null mice were used to monitor the permeability of cell monolayers, the organization of cell–cell junctions and of actin and vimentin networks, and their resistance to shear stress and stretching. The main consequence of plectin deficiency in endothelial cells was the failure of vimentin filaments to attach to focal adhesions, paralleled by an increase in actin stress fibers and increased cellular contractility. The irregular distribution and morphology of actin stress fibers in plectin-deficient cells was confirmed at the ultrastructural level where abundant, perpendicularly orientated actin stress fibers, ending at cell–cell junctions could be visualized [31]. It has been shown that vimentin filaments attach to focal adhesions via plectin and, once anchored, become organized in a cage-like structure that encapsulates the nucleus and stabilizes cortical actin [33,34]. In this way a balance is achieved between the contractile forces exerted by the actomyosin system and the cellular adhesive forces exerted by cortical actin and vimentin. The need of such a balance for the proper functioning of the vascular barrier has been formulated [35]. In all, the ablation of plectin in endothelial cells results in a more leaky vascular barrier and reduced mechanical resilience to shear stress and mechanical stretch [31]. In accordance, plectin-deficient patients have often presented with generalized hemorrhagic blisters [36,37,38,39] and others.

### 3.4. Plectin Ablation in Schwann Cells

The idea that plectin might be important for Schwann cell functions arose because plectin is strongly expressed in myelinating Schwann cells (SCs) [40], and previous studied on muscle had identified plectin as a direct interaction partner of β-dystroglycan [41]. As the major laminin receptor expressed in SCs, α/β dystroglycan (DG) forms a complex that provides a scaffold for proteins required for myelin stability [42]. A Schwann cell-restricted plectin knockout mouse, **P0-Cre/cKO** (plec^loxP/loxP^:Mpz-Cre), was created by breeding plectin floxed mice with P0-Cre mice [43]. In this case, the Cre recombinase is driven by the promoter of the myelin protein zero, a major component of the myelin sheath in peripheral nerves [44]. Plectin-null SCs do not show defects in myelin sheath formation, but the tight association of the DG complex with the intermediate filament cytoskeleton along the Cajal bands is lost. In aging mice, this leads to an increase in the number of axons with myelin sheath deformations. Data gained from the biochemical analysis of primary SCs isolated from these mice showed that P1, P1c, and P1f, together with their rodless versions, are the major isoforms of plectin expressed in SCs, and that plectin interacts with β-dystroglycan and utrophin but not with integrin α6β4, the other laminin receptor, which together with integrin α6β1 is expressed in SCs [45]. Note that P1a, the isoform that binds to integrin β4 in HDs [46], is not expressed in SCs, while isoform P1f takes over the job of binding to β-dystroglycan. However, in the absence of plectin an upregulation of integrin β4 takes place, presumably to compensate for a debilitated DG complex [43]. This could explain the relatively mild effect of plectin deficiency on myelin stability in SCs.

### 3.5. Plectin Ablation in Skeletal and Heart Muscle

Plectin is of crucial importance for skeletal muscle where it connects desmin intermediate filaments to costameres, sarcomeric Z-disks, mitochondria, and nuclei [7,47,48,49]. To better understand the contribution of plectin to the stability and performance of this tissue two muscle-specific transgenic mice were generated by crossing plectin floxed mice with either MCK-Cre [50] or Pax7-Cre mice [51], in which the Cre recombinase is under the control of the muscle creatine kinase promoter or the Pax7 promoter, respectively. **MCK-Cre/cKO** (plec^loxP/loxP^:Ckm-Cre) mice, in which plectin is deleted in striated muscle, developed progressive degenerative alterations of skeletal muscle fibers, leading to reduced endurance, loss of muscle mass, kyphosis, and early mortality [52]. On the tissue level, the absence of plectin resulted in the detachment/loss of desmin IFs from Z-disks, costameres, mitochondria, and nuclei, leading to the formation of desmin aggregates, Z-disk misalignments, detachment of the contractile apparatus from the sarcolemma, and decreased numbers and impaired functions of mitochondria [52]. These phenotypes mimicked the late onset muscular dystrophy characteristics of EBS-MD [10]. Immortalized myoblasts isolated from these mice were used to develop a cell line that could undergo differentiation to functional (contractile) multinucleated myotubes, displaying the pathological features of myofibers, including desmin aggregates and sarcomeric alterations [53]. This cell line was used to identify 4-phenylbutyrate, a chemical chaperon, which alleviated pathological protein aggregation in myofibers and myotubes, and improved muscle performance in vivo [53]. Similar to skeletal muscle, heart muscle showed structural alterations with signs of cardiomyocyte degeneration, as indicated by a large infiltration of connective tissue, misaligned Z-disks, and visible desmin aggregates [52]. There are only a few reported cases of EBS-MD patients with diagnosed cardiomyopathies and arrhythmias [54,55,56,57]. Because cardiac disease is initially asymptomatic and activity dependent, and activity is negligible in patients with MD, the disease might have gone undetected in many cases [55]. A genome-wide association study performed with 14,225 subjects with atrial fibrillation and 374,939 control subjects from the Icelandic population, identified a plectin sequence variant (rs373243633) corresponding to pG3988S, as a moderate risk factor for atrial fibrillation [58].

Evidence that plectin is involved in the formation and integrity of the neuromuscular junction comes from patients with loss-of-function mutations who develop EBS-MD with congenital myasthenic syndrome (EBS-MD-MyS) [10] (see also below). Congenital myasthenic syndrome is known to be caused by defective synaptic transmission at the neuromuscular junction (NMJ) [59,60]. To assess the effects of plectin deficiency on NMJ integrity, a second conditional mouse model, **Pax7-Cre/cKO** (plec^loxP/loxP^:Pax7-Cre), was generated that enables plectin inactivation in skeletal muscle precursor (satellite) cells, and consequently minimizes the effects of plectin renewal in adult cells through satellite cells [51]. Pax7-Cre/cKO mice showed progressive weakness, profound kyphosis and a reduced survival rate already at an early age [61]. Proper function of the NMJs requires presynaptic release of acetylcholine from the motor nerve terminal and the clustering of postsynaptic acetylcholine receptors (AChRs) on the muscle plasma membrane to trigger muscle contraction. However, in the skeletal muscle of Pax7-Cre/cKO mice, the endplates, the postsynaptic part of the NMJ, were fragmented into small islands of AChRs, postsynaptic infoldings were strikingly rare and of uneven shape, and normally abundant local mitochondria were scarce; there was also a massive invasion of microtubules into the postsynaptic local area [61]. In contrast to wild-type mice where dense desmin IFs networks were observed juxtaposed to the NMJ, in Pax7-Cre/cKO mice the networks were detached from synapses and collapsed filaments formed aggregates around synaptic nuclei. Ex vivo analyses of cultured myotubes, differentiated from immortalized plectin-deficient myoblasts [53], confirmed these results, showing that AChRs were unable to coalesce into stable clusters and lacked IF attachment. These defects could be reversed by the forced expression of plectin isoform P1f but not by nuclear/ER membrane-associated isoform P1 or Z-disk-associated isoform P1d, validating the specificity of P1f–NMJ interaction. At the molecular level, desmin IFs were shown to become attached to the NMJ via P1f’s direct interaction with the AChR-scaffolding protein rapsyn. AChR cluster fragmentation and disorganization of the NMJ have also been reported for desmin-null mice [62,63] and double mdx/desmin KO mice [64]. Functional tests directed to quantify the performance of muscle strength, limb coordination, and locomotion activity in the Pax7-Cre/cKO mice, pointed to a significant reduction in all of the parameters tested when compared to wild-type mice. In summary, the Pax7-Cre/cKO mouse model showed that plectin anchors desmin IF networks at the NMJ and, thereby, promotes the formation and stabilization of AChR clusters and of the invaginating postsynaptic folds [61].

Plectin was found to be overexpressed at the sarcolemma of myofibers from dystrophin-deficient MD patients [65] as well as from the corresponding mdx mouse model [41]. As shown for mdx mice, the accumulation of plectin to the sarcolemma is due to the recruitment of isoform P1f to a domain of β-dystroglycan that normally is occupied by dystrophin [41]. To test whether the higher level of plectin could compensate for the loss of dystrophin by stabilizing costameres and the sarcolemma-associated cytoskeleton, a **plectin/dystrophin double KO** (**dKO**) mouse line was generated by crossing MCK-Cre/cKO with mdx mice. The comparative phenotypic analyses of the dKO, wild-type, mdx, and MCK-Cres/cKO mice, revealed that the ablation of plectin in mdx mice indeed restored sarcolemmal integrity and glucose uptake to normal levels, although it drastically reduced the lifespan and worsened the overall phenotype of the mice [66]. Previous work has shown that mdx mice have an increased body weight coupled with a deficient energy metabolism [67]. Raith et al. [66] extended these findings by demonstrating that the reduced glucose uptake recorded in mdx mice was not due to insufficient insulin secretion or insulin-independent signaling but to a compromised translocation of the glucose transporter GLUT4. The authors suggested that in mdx myofibers, plectin acts as a local antagonist of MT network formation, thereby hindering MT-dependent delivery of GLUT4 to the membrane [66].

## 4. Isoform-Specific Plectin KOs

**Figure 2:**
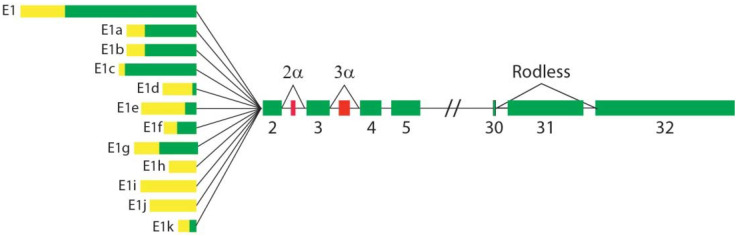
Plectin transcripts generated by alternative splicing. Twelve first exons splicing into exon 2 are shown. Untranslated regions (yellow) and coding regions (green) are marked. Two optionally spliced exons, 2α and 3α, inserted between exons 2 and 3 and 3 and 4, expressed in some isoforms, are marked in red. Alternative splicing of exon 31 encoding plectin’s rod domain generates the rodless isoform. The figure is not drawn to scale.

All four isoform-specific KO lines described below were generated by homologous recombination, targeting one single plectin isoform. Note that in these mouse lines, the chosen isoform is inactivated across all tissues, whereas in the tissue-specific (conditional) KO lines described above all isoforms are inactivated in only one tissue. Plectin isoforms (Figure 2) were named in chronological order of their identification as 1, 1a, 1b, 1c, etc. [3,4]. In contrast, transcript variants were termed in numerical order [68] according to their position in the genome [4]. Unfortunately, this has led to the paradox that plectin isoform 1 corresponds to transcript variant 6, transcript variant 1 corresponds to plectin isoform 1c, etc. (see Figure 1c in reference [1]). To monitor the expression of different isoforms, several isoform-specific antibodies, using the corresponding specific *N*-terminal peptides as immunogen, were raised in rabbits [69].

### 4.1. Plectin Isoform 1 (P1)-Deficient Mice: P1-KO

P1 has the longest isoform-specific sequence (180 amino acid residues) among all the isoforms. Besides containing binding sites for Siah ubiquitin ligase [70], endophilin B2 [71], and β-synemin [72], and numerous positively charged amino acid residues that could attract negatively charged moieties of other molecules, it also contains a classical nuclear localization signal [73]. Early studies designed to determine the subcellular localization of the isoforms revealed that only short, truncated *N*-terminal versions of P1 were imported into the nucleus, while full-length P1 remained in the cytoplasm in perinuclear regions [6]. In a later report, plectin was identified as a binding partner of the outer nuclear protein, nesprin-3a, to which it binds via its *N*-terminal ABD domain [74]. As nesprin-3a binds to the inner nuclear membrane proteins, SUN1 and SUN2, which extend their N-termini into the nucleoplasm, a link between the nucleus and IFs via plectin is established [75]. However, the phenotypic analysis of a nesprin-3 KO mouse only supports this model for a subset of Sertori cells of the testis, where nesprin-3 is required for the localization of both plectin and vimentin at the nuclear perimeter [76]. In a study focused on the role of P1 in muscle [77], it was shown that in P1-deficient myofibers, desmin networks were disorganized, having collapsed and formed aggregates in the perinuclear areas. Moreover, the myonuclei were no longer linearly arranged and regularly spaced, had lost their typical ellipsoidal shape, and their capacity to translocate along the long myofiber axis was drastically reduced. These alterations in nuclear morphology and mobility were paralleled by changes in chromatin organization and gene expression, especially genes involved in mechanotransduction, such as the transcription factors YAP/TAZ, STAT1, and STAT3, and members of the MAP kinase pathway, ERK1 and ERK2, showed significantly reduced expression [77]. As in plectin-null myotubes [53], heat shock protein hsp27 was strongly upregulated, which combined with its colocalization with perinuclear desmin aggregates, is indicative of a massive chaperone activation in response to IF network collapse. P1 docking onto the outer nuclear membrane was shown to take place via endophilin B2, a BAR domain-containing protein that binds to a short peptide motif within the isoform-specific head domain of P1. In summary, this study shows that P1 targets and anchors the desmin filament network to the outer myonuclear membrane, regulates the shape and regular distribution of nuclei along the myofiber, reinforces the connection between the nucleus and the cytoskeleton, and, in this way, directly affects the mechanotransduction machinery [77].

P1 is prominently expressed in tissues of mesenchymal origin, such as connective tissue and muscle [4]. Immunostaining of cryosectioned mouse tissues and immunoblotting of cell lysates, using P1-specific antibodies, combined with RT-PCR, revealed expression of P1 in dermal fibroblasts and dendritic cells of the epidermis and in tissues of immunological origin such as the lymph nodes, spleen, thymus, primary T cells, and macrophages [78]. Mice deficient in P1 had no skin blistering, but their wound healing was affected as leukocyte infiltration was significantly reduced. P1-deficient primary fibroblasts derived from these mice showed normal tubulin and vimentin distribution but alterations in the actin cytoskeleton; similarly, cultured T cells did not show any morphological alterations but their ability to polarize and form uropods was compromised; moreover, the migration potential of both dermal fibroblasts and T cells was significantly impaired [78].

### 4.2. Plectin Isoform 1b (P1b)-Deficient Mice: P1b-KO

In the short *N*-terminal P1b-specific sequence (37 amino acid residues), there is a mitochondria targeting signal [79] that targets P1b to mitochondria and anchors it in their outer membrane [80]. Plectin’s association with mitochondria and desmin networks was first demonstrated on the ultrastructural level using double immunogold labelling [48]. A first hint to the isoform identity was provided in [6], while the final proof for P1b mediating the coupling of mitochondria to IFs came from the analysis of the P1b-KO mouse [52,80,81]. P1b-KO mice showed a pathological muscle phenotype characterized by altered morphology and reduced numbers of mitochondria, disorganized mitochondrial networks, detachment of mitochondria from the desmin cytoskeleton and Z-disk structures, and the presence of large mitochondrial aggregates in subsarcolemmal regions, ultimately resulting in myofiber degeneration. Other alterations included reduced respiratory activity, most pronounced in the heart, soleus, and gastrocnemius, and a significant decrease in the apparent Km for adenosine diphosphate (ADP) as well as an increased outer mitochondrial membrane permeability [81]. An association of plectin with VDAC (voltage dependent anion channel, also known as porin), reflected by an increased permeability of the outer mitochondrial membrane, points to a plausible participation of P1b in the metabolic crosstalk between the mitochondria and the rest of the cell [82,83]. Mitochondria in P1b-deficient fibroblasts showed substantial changes in shape, elongation, and network formation but their motility was unaffected [80]. The morphological alterations of mitochondria were associated with a deregulation of the mitochondrial fusion–fission machinery, specifically an upregulation of mitofusin-2, a mitochondrial fusion protein [84,85]; an elongated shape of mitochondria is characteristic also of plectin-null myoblasts [53]. Mitochondrial alterations have frequently been observed in skeletal muscle biopsies from EBD-MD patients [10]. Particularly noteworthy is the case of a patient with severe mitochondrial dysfunctions as part of a multisystem disorder affecting skeletal muscle, skin, heart, eyes, and brain. The mutation in this case led to the expression of a truncated protein missing the C-terminal tail of plectin [86].

### 4.3. Plectin Isoform 1d (P1d)-Deficient Mice: P1d-KO

Plectin association with Z-disks of striated muscle is well documented and supported by data from EBS-MD patients and plectin KO mice [7,10,13,15]. After the first report on plectin localization at Z-disks [7], several studies demonstrated plectin to associate with the periphery of Z-disks, linking desmin filaments to it and interlinking myofibrils; furthermore, the plectin–desmin bond was shown to start at premyofibrillar stages and persist during myocyte differentiation [47,87,88]. The identification of P1d as the isoform specifically associating with Z-disks was reported in [41]. When P1d-KO and other isoform-specific KO mouse lines became available, a comparison of immunolabeled cryosections of muscles revealed regular Z-disk arrays extending to the sarcolemma in all of the knockouts except for the P1d-KO. Notably, in desmin KO mice, plectin was properly localized at Z-disks [89,90], indicating that Z-disk association of plectin is desmin filament-independent. P1d-KO mice showed all of the characteristics of myofibrillar myopathies including muscle degeneration, misalignment of costameres, and accumulation of desmin/synemin aggregates predominantly around interior Z-disks, along with the loss and lysis of mitochondria and decreased respiratory activity. It has been reported that synemin and syncoilin anchor desmin IFs to Z-disks and costameres via their interaction with α-actinin and vinculin [91,92]. However, in the absence of plectin these interactions apparently are too weak to sustain the attachment of desmin at these sites, as both proteins were found to be co-aggregating with desmin [52,81]. The muscle phenotypes of plectin P1d-KO and desmin KO mice were quite similar but only partially overlapped, as desmin in muscle is associated also with other plectin isoforms that are not affected in the P1d-KO [52]. The combined deficiency of P1d and desmin in the double **P1d/desmin KO** resulted in a more hefty muscular damage, manifesting as decreased body weight, lifespan, and muscle strength. From these data it can be concluded that P1d maintains the integrity of the muscle fiber at the level of the Z-disk by anchoring desmin to adjacent myofibrils and connecting the contractile apparatus to costamere-associated desmin IFs [52].

### 4.4. Plectin Isoform 1c (P1c)-Deficient Mice: P1c-KO

Early studies of plectin in the nervous system showed expression in the brain and spinal cord, including ependymal cells, astrocytes, motor neurons, and others [93,94], but it was only after the identification and expression analysis of the different plectin isoforms that P1c was identified as the dominant isoform expressed in neural tissues [4], followed by first evidence for an association with microtubules [6,17]. Data gathered with P1c isoform-specific antibodies showed a polarized distribution of P1c to the postsynaptic dendritic cell compartment of non-myelinated central nervous system neurons [40]. In ependymal cells and astrocytes, other plectin isoforms were expressed in addition to P1c [40], e.g., isoform P1e is second to P1c in cortical astrocytes (Wiche, unpublished data). P1c is also abundantly expressed in the suprabasal layers of the epidermis [17,95]. Plectin interaction with MTs and MT-associated proteins (MAPs) was demonstrated in several in vitro assays [96,97], but it was the seminal publication of G. Borisy’s group [98], visualizing plectin molecules as thin (2–3 nm) and up to 200 nm long filaments bridging MTs with vimentin filaments, that provided the first convincing evidence that this interaction occurs also in the cells. In the brain, it has been shown that P1c, in combination with two short exons, 2α and 3α, inserted into the ABD of plectin, targets plectin to MTs [99]. A P1c-KO mouse line was generated to study the function of isoform P1c in vivo [40]. Mice of this type had a normal life span without showing any visible phenotype. A more in-depth phenotypical analysis revealed reduced nerve conduction velocities in sciatic motor neurons but normal velocities in sensory neurons. In general, axons did not show myelination defects or gross structural abnormalities, although a trend towards axons with smaller diameters and larger neurofilament spacing was observed [40]. When dorsal root ganglia and hippocampal neurons derived from P1c-KO mice were analyzed, alterations in neuritogenesis, growth cone morphology, and the movement of vesicles and mitochondria could be demonstrated [99]. In behavioral tests, P1c-KO mice showed impaired pain sensitivity, reduced fear conditioned memory, and diminished long-term memory [99].

The 5′ untranslated region of P1c is quite long and includes three non-coding exons that are alternatively spliced into exon 1c [40]. Within this region, P1c overlaps with the 3′ end of poly(ADP-ribose) polymerase (PARP) 10 gene in a head-to-tail arrangement but without readthrough transcription from the PARP10 gene into P1c sequences [100]. The generation and analysis of a conditional mouse model, **Nes-Cre/cKO**, where plectin was knocked down but PARP10 was unaffected, confirmed that the phenotypes described above were due exclusively to P1c depletion [40]. Nes-Cre/cKO is a neuronal cell-restricted conditional mouse line, devoid of plectin in neural tissues due to the removal of the floxed plectin allele by a cre recombinase driven by the nestin promoter [101]. Recently, a region of chromosome 8q24.3, which includes plectin, has been identified as a risk locus for osteoarthritis [102]. The osteoarthritis risk-conferring allele rs11780978 (G > A), located upstream of the exon P1c coding region, correlated with reduced plectin expression due to methylation at a cluster of nine CpGs [103].

### 4.5. Human Knockouts for Isoforms P1a and P1f

A few humans carrying plectin mutations in alternative first exons of the plectin gene have been identified. These mutations are of great interest because the affected isoform dictates disease manifestation, while the other isoforms are expressed at normal levels. In one of these cases, a homozygous nonsense mutation (c.46C > T; p.Arg16X) located in the isoform-specifying first exon of P1a, was identified in two sisters born to consanguineous parents. In contrast to EBS-MD patients who carry mutations in regions of the plectin gene that are common to all isoforms, these patients had a skin-only phenotype, characterized by generalized blistering, excoriated verrucous papules, blisters healing with scarring and hyperpigmentation, and dystrophic hand and toe nails, while mucous membranes, heart, and muscle were spared [104]. As an integral part of HDs, P1a associates with integrin α6β4 and together with BPAG1e links the keratin cytoskeleton of basal keratinocytes to the extracellular matrix [17,46,95]. Accordingly, skin biopsies of the patients showed hypoplastic HDs [104]. A dominant mutation in the plectin gene that leads to the selective degradation of P1a in keratinocytes and causes a skin-only phenotype known as EBS-Ogna [95] will be discussed below.

In another instance, two different homozygous mutations have been identified that abolish the expression of isoform P1f. One of them consisted of a small deletion that eliminated the start codon of P1f, the other of a nonsense mutation within codon 20 of exon 1f. In the first case, several members of three unrelated families, but originating from neighboring cities, carried a homozygous 9 bp deletion at the 5′ end of exon 1f (c.1_9del; p.Met1_Gly3del; reference sequence NM_201378.3) [105]. The affected individuals, showing generalized proximal muscle weakness without apparent myasthenic features or skin blistering, have been diagnosed as having limb-girdle muscular dystrophy type 2 (LGMD2), now renamed LGMDR17 (MIM#613723). The phenotype manifests already in early childhood, progresses slowly until the late teens, but rapidly later on [105]. A later report described the phenotype of four new homozygous carriers of the same c.1_9del deletion, belonging to four unrelated families, all of them consanguineous and originating from the same region as the previous cases, suggesting a common origin of the mutation. These patients presented with slowly progressive proximal muscular weakness, fatigue, and muscle cramps similar to the patients reported by Gundesli et al. [105]; however, additionally, they showed clear myasthenic symptoms such as ptosis, nasal speech, tongue weakness, neck flexor and paraspinal muscle weakness, and mild scoliosis, with electromyographies showing myopathic changes; but, their skin was spared [106]. The second mutation identified in exon 1f, c.58G > T, p.Glu20X (reference sequence NM_201378.3), caused LGMDR17. The affected individuals, three siblings, presented muscular dystrophy aggravated by respiratory problems, mainly atelectasis and dyspnea; two of the siblings died because of lung collapse [107]. As the reported lung phenotype had never been diagnosed in plectin-deficient patients or mice, although plectin is prominently expressed in alveolar epithelial cells [108], it seems that neither the patients nor the mice were thoroughly examined for pulmonary defects. Another possibility is that the lung phenotype was caused by a second unidentified mutation. In summary, all P1f-deficient patients suffer from LGMDR17, and probably also from myasthenia, but none of them show skin involvement. P1f is strongly expressed at the sarcolemma where it organizes the sarcomeric lattice [41] and attaches the contractile apparatus to the sarcolemma via the desmin IF cytoskeleton [52]. Accordingly, muscle biopsies of all affected individuals with the c.1–9del or c.58G > T mutation showed sarcolemma fragmentation, Z-disk disorganization, and disruption of myofibrils and mitochondria. As P1f has been shown to play a crucial role in the maintenance of NMJ integrity by recruiting and connecting IFs to the postsynaptic domain [61], it is not unexpected that P1f-deficient patients present with myasthenic symptoms; however, in many cases, myasthenic features have probably been underestimated. All in all, the phenotypes of the patients described here support the general concept that each plectin isoform performs a specific function(s), and that P1a and P1f play key roles in skin and skeletal muscle.

**Figure 3:**
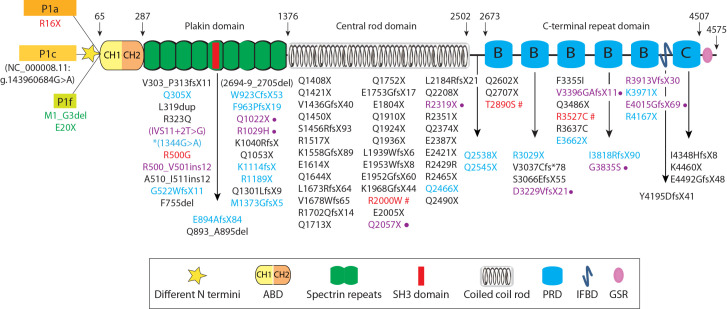
Schematic representation of the plectin molecule showing its structural organization and the localization of disease-causing mutations. The central α-helical rod domain, which forms a coiled-coil structure upon dimerization, and the subdomains of the flanking N- and C-terminal globular domains are indicated. Note that the plakin domain consists of 9 spectrin repeats with an SH3 domain embedded in repeat 5. The 6 plectin repeat domains at the C terminus are of the B and C type (for details see Introduction). Numbers above the molecule indicate the amino acid residue positions at the domain borders. ABD (actin binding domain), PRD (plectin repeat domain), IFBD (intermediate filament binding domain), GSR (Gly-Ser-Arg repeats). Plectin mutations are annotated according to the convection “Mutations in the plectin gene related to human diseases should be named based on the position in NM_000445 (variant 1, isoform 1c), unless the mutation is located within one of the other alternative first exons, in which case the position in the respective Reference Sequence should be used (provided by RefSeq, August 2011)” even when this was not followed in the original publication. Mutations causing epidermolysis bullosa (EBS) with muscular dystrophy (EBS-MD) are listed in black, EBS with pyloric atresia (EBS-PA) in blue, EBS with limb-girdle muscular dystrophy (EBS-LGMD) in magenta, and EBS-MD with congenital myasthenic syndrome (EBS-MD-Mys) in magenta followed by a dot, LGMD autosomal recessive 17 (LGMDR17) in green, EBS and EBS-Ogna in red, the hashtag indicates autosomal dominant.

An overview of the plectin mutations reported thus far is presented in Figure 3. Plectin mutations are spread over the entire gene, and their phenotypes probably reflect their location in the different domains of the molecule. However, a clear correlation between the position of a mutation and a disease type has not come forward yet. The pattern of inheritance is autosomal recessive, with just a few exceptions (see Figure 3). Five different genetic disease phenotypes have been recorded for plectin mutations in Mendelian Inheritance in Man (OMIM) under the entry MIM #601282: EBS-MD, EBS with pyloric atresia (EBS-PA), LGMDR17, EBS-Ogna, and EBS with nail dystrophy (EBS-ND). Details of the respective phenotypes can be found in the different MIM entries given below and in [10]. The first and most frequently identified mutations in the plectin gene were found in the central rod domain. Individuals carrying homozygous or compound heterozygous mutations in this location suffer from EBS-MD, which is characterized by skin blistering and progressive muscular dystrophy (MIM #22670). Similar to plectin-null, MCK-Cre/cKO, P1-, P1b-, and P1d-KO mice, muscle specimens from these patients showed myofibrillar degeneration, disruption of the desmin networks with formation of desmin aggregates, clustering of nuclei, mitochondrial abnormalities, and symptoms of cardiomyopathy. Among the mutations in the N- and C-terminal domains of plectin some cause EBS-PA and others LGMDR17 or EBS-MD-Mys. EBS-PA is characterized by severe skin blistering and congenital pyloric atresia (MIM #612138). Because EBS-PA is most commonly caused by mutation in the integrin β4 gene, it is believed that the mutations causing EBS-PA alter contact sites of plectin with integrin β4, abolishing their interaction. LGMDR17 is characterized by early childhood onset of proximal muscle weakness and atrophy without skin involvement (MIM #613723). Mutant phenotypes reported as EBS-LGMD formally fall under the category of EBS-MD, while EBS-MD-Mys refers to EB-MD with congenital myasthenic syndromes, which include neuromuscular pathologies affecting the muscles of the face, neck, throat, eyes, and limbs. Additionally reported symptoms described for EBS-MD patients comprise respiratory, cardiac, and urogenital complications. EBS-Ogna (MIM #131950) and two other mutations with a skin-only phenotype are autosomal dominant. Plectin genetic diseases (plectinopathies) are classified as rare diseases.

## 5. Plectin Knock-In Mouse Models

Knock-ins create a one-for-one substitution of the DNA sequence within the genome, for example, introducing a point mutation in one gene to mimic a human disease, or exchanging the mouse endogenous gene for its human counterpart. In the case of plectin, this technology has been used to study the EBS-Ogna variant and rodless plectin, an isoform missing the rod domain.

### 5.1. EBS-Ogna Mice

Most mutations in the plectin gene are inherited in an autosomal recessive fashion resulting most commonly in EBS-MD but also in other types of EBS [10]. In contrast, EBS-Ogna is caused by an autosomal dominant mutation consisting of a C > T transition in the exon coding for the rod domain of plectin (c.5998C > T, p.R2000W; RefSeq NM_000445.3) [109]. This form of EBS was identified in a large Norwegian kindred living in the town of Ogna [110], but it is not restricted to this kindred as individuals from nine apparently unrelated families of Norwegian, German, Dutch, and Turkish descent have been identified as carriers [37,109,111]. The skin phenotype of the patients is relatively mild, being characterized by generalized epidermal fragility, trauma-induced acral blistering, bruising, erosions, and small blood blebs. Whereas the immunolocalization of plectin in the epidermis of control samples showed strong intracellular staining of suprabasal as well as basal keratinocytes, virtually no reactivity was found in the basal keratinocytes of the patients. However, the outstanding feature of these patients is the absence of a muscle phenotype [109]. To answer the question of how a missense mutation in a plectin domain common to all isoforms results in a skin-only phenotype, a knock-in mouse was generated, which integrated the human Ogna mutation in exon 31 into the plectin locus. Reproducing the key features of Ogna patients, Ogna mice (plec^Ogna/+^) showed skin fragility, sparse and hypoplastic HDs with impaired keratin filament association, and no development of muscular dystrophy [95]. Immunostaining of skin sections using isoform-specific antibodies revealed that, except for a few positive patches, no expression of plectin’s HD-associated isoform P1a was observed in the epidermis of mutant mice; in contrast, expression of P1c was well preserved in the mutant tissues. In vitro biochemical studies performed with a purified recombinant version of the rod domain and in silico protein modeling suggested that the mutation causes a local disruption of the α-helical coiled-coil, having only a minor structural impact on the rod structure as a whole. This was also reflected in just a minor difference in the midpoints of dissociation kinetics, which is common for mutants in long coiled-coils with a single amino acid substitution [112]. The 3D modeling suggested that the p.R2000 W mutation abolished the electrostatic interaction between R2000 and E1993, concomitantly exposing the hydrophobic side chain of tryptophan to the solvent. As this situation is energetically unfavorable, it was hypothesized that the W2000 side chain enters into the apolar inter-helical interface, leading to a local unfolding of the helix and disruption of the coiled-coil while the rest of the rod domain is unaffected.

On a more mechanistic level, the study of Walko et al. [95] showed that the local conformational change renders the mutant protein more vulnerable to cleavage by calpain-1 and other proteases that are activated specifically in the epidermis but not, for example, in skeletal muscle. Calpain-1 is localized in the granular and basal layers of the skin, suggesting a close spatial relationship of calpain-1 and P1a in vivo. Ex vivo, ionomycin-induced activation of calpain-1 caused the degradation of P1a and integrin β4, and led to the progressive destruction of HD-like protein complexes, a process that could be partially blocked by the application of calpain inhibitors. Although isoforms P1a and P1c both are expressed in basal keratinocytes, only P1a as part of the HD was susceptible to degradation, suggesting that the proteolytic degradation of P1a was site-specific. Analysis of P1a expression levels in mutant mouse tail skin by semiquantitative confocal immunofluorescence microscopy [113] revealed that plectin expression in Ogna epidermis is well below the level measured for heterozygous (plec+/−) mice. As such, these mice, as well as heterozygous human carriers of plectin mutations, do not display a skin pathology; the amount of plectin expressed in their epidermis defines the type of threshold required for plectin functionality. Not reaching this threshold, epidermal P1a levels in Ogna mice were considered as too low to support the formation of HDs in sufficient numbers for stable keratin filament anchorage, ultimately resulting in skin fragility in response to mechanical stress [95].

### 5.2. Rodless Plectin Mice

The rodless isoform of plectin was already detected at the time when plectin was first cloned from a rat glioma C6 cell cDNA library [114], but it was not identified as a variant arising from alternative splicing of a single exon (exon 31) encoding the entire rod domain of plectin, until the exon intron structure of the rat plectin gene was established [3]. Rodless transcripts and proteins were identified in various rat tissues [3], human skeletal muscle [49,56,86,88], and mouse brain [115]. Additionally, they were detected in primary and immortalized fibroblasts, keratinocytes, and amniocytes derived from patients suffering from EBS-MD or junctional epidermolysis bullosa with pyloric atresia, and the corresponding healthy controls [86,116,117,118]. As it has been hypothesized that the rodless variant might partially rescue the phenotype of human carriers of plectin mutations [117,119,120], a knock-in mouse line (rodless) was generated that expressed rodless but no full-length plectin (Plec^ΔEx31/^^ΔEx31^) [121].

Rodless mice have a normal life span, develop normally, and show no signs of skin blistering, muscular dystrophy, or other gross abnormalities. No significant changes were observed in HD structure, composition, or density, albeit, mutant mouse-derived keratinocyte showed an increase in their migration rate [121], similar to keratinocytes derived from plectin-null or Ogna mice [95,122]. On skeletal muscle sections, plectin, β-dystroglycan, and integrin β1 showed normal localization, but subtle changes in mitochondria or Z-disk alignment were not investigated. However, no signs of muscle weakness were observed in rodless plectin mice, not even after intensive running, or at old age [121]. Biochemically, it was shown that the rodless isoform can dimerize, presumably via the ABD or a region at the beginning of the C-terminal domain, with the potential to form a coiled-coil. At the cellular level, it was shown that the distance between plectin’s keratin-binding C terminus and integrin β4 (to which it binds via its N-terminus) is shorter in rodless plectin-expressing keratinocytes than in full-length plectin-expressing wild-type cells [121]. This shorter distance seemed not to have repercussions on the functionality of HDs in vivo, although assays to check skin fragility of the mutant mice were not performed. Interestingly, rodless plectin was expressed in rodless mice at much higher levels (of both mRNA and protein) than in mice expressing also the full-length variants, a result that seems to support the hypothesis of functional compensation [121]. However, the human situation might be different, as the rodless isoform is far less abundant than full-length plectin [3,49,56,88,115,117,118]. Available data based on semiquantitative RT-PCR indicate full-length/rodless ratios of ~14 and ~21 for fibroblasts and keratinocytes, respectively [117]; in mouse brain the ratio was ~20 [115]. Data from patient specimens are rare, but, in the few reported cases, visual inspection (without quantification) showed very low expression of the rodless isoform [56,86,117]. To reconcile the hypothesis of rodless variants compensating for the lack of the full-length isoforms with the data from the mouse model, and with the fact that patients develop the disease apparently without upregulation of the rodless variant, it has been postulated that functional compensation ultimately will depend on the expression level reached by the rodless isoform in each case [121]. In this context, the detailed data presented in Winter et al. [56] are very illustrative as they allow a direct comparison between the genetic mutation and its location as well as rodless versus full-length expression, and the clinical phenotype for three cases of EBS-MD (see Table 1 and Figure 2b,c in reference [56]). However, the clinical history of one of the patients who has a high level of rodless plectin hardly supports the concept that rodless plectin can compensate for of the lack of full-length plectin [56].

## 6. Zebrafish and *C. elegans*

Apart from mice, plectin has been studied in the fish *Danio rerio*, known as zebrafish and *C. elegans*. Zebrafish is very well suited to the study of muscle development and muscle diseases, as the genes encoding structural and regulatory components of the contractile apparatus, as well as sarcomere and costamere-associated proteins, are highly conserved, assays to assess muscle performance and neuromuscular function are fast and easy to evaluate, and structural alterations of the muscles are readily detected by birefringence measurements. Zebrafish often have duplicated genes (paralogs) to a single mammalian gene. In the case of plectin they are named plectin a and plectin b and are located in chromosome 19 and 16, respectively [123]. Expression of plectin has been detected as early as 1 day post fertilization (dpf) in the yolk syncytial layer, with increasing levels at later stages. From the pharyngula stage (2–3 dpf) onwards, plectin was detected in the epidermis [124,125], while data for muscles are not available. Knock-down of the plectin genes via morpholino-modified antisense oligonucleotides, injected at the one-cell stage and analyzed at consecutive developmental stages, showed progressively impaired voluntary mobility and slow touch escape response, myofibrillar disorganization and degeneration, and mitochondrial abnormalities; paradoxically, protein aggregates were not observed. Additionally, the mutants exhibited diminished cardiac contractility or arrhythmias [126].

*C. elegans* is a common model organism for studying developmental biology including embryogenesis. One of its advantages lies in its anatomical simplicity and its transparent outer cuticle that allows observers to see the internal structures and to follow cell lineage. The plectin ortholog in *C. elegans*, Vab-10 A, is encoded by the gene *Vab-10* that, by alternative splicing, produces two isoforms, Vab-10 A/plectin and Vab-10B/MACF1a. Both isoforms share a common amino-terminal half, whereas the carboxy terminus is unique for each isoform [127]. Expression of Vab-10 A is detected early in embryogenesis at the epidermal–muscle interface connecting the apical extracellular matrix to the basal extracellular matrix, in what appears as an adhesion complex called fibrous organelles [128]. These HD-like structures (CeHDs) are formed by a backbone of IFs bridged by Vab-10 A at each end and attached to the apical side through the transmembrane receptors MUP-4 and MUA-3, and to the basal side through LET-805, the *C. elegans* equivalent to integrin β4 in vertebrate HDs [127,129,130]. Loss of VAB-10A halts embryonic elongation from the two-fold stage onwards and causes the detachment of muscle cells from the epidermis and their collapse inside the embryo [127]. It was shown that in a double Vab-10A/PAK-1 kinase mutant background, CeHDs cannot maintain their integrity when exposed to muscle-induced tension, while in a wild-type background and in concerted action with signaling molecules found at the CeHDs, they can activate a mechanotransduction pathway in response to tension exerted by muscle contractions [131]. The region mediating mechanotransduction has been narrowed down to the SH3 and flanking spectrin repeats of Vab-10 A [132]. Vab-10 A, the only evolutionary conserved CeHD protein, has a plakin domain consisting of nine SR with an atypical SH3 domain embedded within the central SR5. Detailed structural studies of plectin’s plakin domain have shown that the SH3 domain is partially masked by intramolecular contact with the adjacent SR4 [133], and it was speculated that force-induced conformational changes coupled with the optional unfolding of individual SR might confer the plakin domain the possibility to transmit tension [134]. In fact, the molecular dynamic simulation showed that tension will unmask the SH3 domain by the unfolding of the SR4 and/or SR5 domains [135]. Accordingly, tension generated on the CeHDs by muscle contractions will expose the SH3 domain, making it available for other protein interactions [132].

## 7. Concluding Remarks

Most of what is known to date about the biological functions of plectin has been gained through the analysis of a series of different knockout mouse models. Although the first null knockout line was established in 1997, it took over a dozen other transgenic lines of different types, to gain an understanding of plectin’s functional diversity and disease mechanisms. Because of the highly complex pattern of alternative plectin gene splicing, it was necessary to resort to isoform-specific and tissue-specific conditional knockout models, supplemented with double knockout models, to individually dissect plectin’s multiple functions. Dissecting these functions is further complicated by difficulties in raising antibodies against the sometimes quite short peptides encoded by the different first exons. As of today, antibodies discriminating between isoforms are available only for a few of the isoforms, and some of the isoforms identified are still waiting to be studied. An example is soform P1e, which together with P1c is prominently expressed in astrocytes. Plectin has been shown to localize to ependymal cells, astrocytes in white matter, small blood vessels, and the astrocytic processes surrounding them [93]. A conditional astrocyte plectin knockout or an isoform-specific knockout could provide interesting information about the functions of P1e. Furthermore, such mice would be amenable to testing cognitive behavior, as growing evidence indicates that astrocytes actively participate in information processing at synapses. *C. elegans*, too, is a powerful system to study the functional characteristics of plectin on the molecular level. Since the identification of Vab-10A as the plectin ortholog of *C. elegans*, and by using the power of forward and reverse genetic screens, M. Labouesse’s group has made significant contributions to establishing the role of plectin in the assembly of muscle attachments during early development, and the involvement of the SH3 domain as a central player in mechanotransduction. Transgenic mice and *C. elegans* are expected to continue delivering new insights into plectin functions, while a takeoff of plectin research in zebrafish is anticipated.

## Data Availability

This review does not present new experimental data. For data availability please refer to the respective original papers.
